# Cost-Efficient and Fast At-Line Assessment of Content and Uniformity in Low-Dose Dimdazenil Capsules Using Transmission Raman Spectroscopy

**DOI:** 10.3390/pharmaceutics18030298

**Published:** 2026-02-27

**Authors:** Xun Ma, Lianlian Shan, Shuangpeng Zhu, Zihan Zhu, Shuyu Lu, Mingzhe Xu, Lihui Yin

**Affiliations:** 1 NMPA Key Laboratory for Quality Research and Evaluation of Chemical Drugs, National Institutes for Food and Drug Control, Beijing 102629, China; maxun@nifdc.org.cn (X.M.); 3322010555@stu.cpu.edu.cn (Z.Z.); 3323010554@stu.cpu.edu.cn (S.L.); 2 Xinjiang Uyghur Autonomous Region Institute for Drug Control, Urumqi 830054, China; 13579808851@163.com; 3 Zhejiang Jingxin Pharmaceutical Co., Ltd., Shaoxing 312500, China; zhushuangpeng@jingxinpharm.com; 4 School of Pharmacy, China Pharmaceutical University, Nanjing 210009, China

**Keywords:** transmission Raman spectroscopy, design of experiments, partial least squares analysis, quantitative analysis, dimdazenil

## Abstract

**Background/Objectives**: Transmission Raman spectroscopy (TRS) is widely used for non-destructive quantification of solid dosage forms, yet most applications involve high-dose formulations (>5% *w*/*w* active pharmaceutical ingredient (API)). This study addresses the underexplored area of low-dose drug analysis by developing a TRS method for dimdazenil capsules containing only ~1.5% *w*/*w* API—a concentration rarely reported in the current TRS literature. **Methods**: A partial least squares (PLS) model was built using TRS spectra of dimdazenil capsules, with sample variability introduced via a design of experiments (DoE) approach. Model performance was validated against reference high-performance liquid chromatography (HPLC) data from independent samples and three commercial batches, incorporating specificity checks and greenness assessment via AGREEprep. **Results**: The TRS method achieved reliable variation assessment with acceptable accuracy, showing relative errors below 5.0% across all validation sets despite a minor systematic bias of ~−2.87%. Analysis required no sample preparation and took less than 150 s per capsule. Specificity was ensured by the unique 1628 cm^−1^ band of dimdazenil, with no interference from excipients confirmed by spectral examination and multivariate statistical methods. The method scored 0.86 on AGREEprep, highlighting its environmental superiority over HPLC. **Conclusions**: This work demonstrates that TRS can be reliably extended to low-dose solid dosage forms (~1.5% *w*/*w*), a concentration range rarely addressed in the existing TRS literature. This significantly broadens its applicability in pharmaceutical quality control and supports its potential integration into continuous manufacturing for challenging low-concentration products.

## 1. Introduction

**Figure 1:**
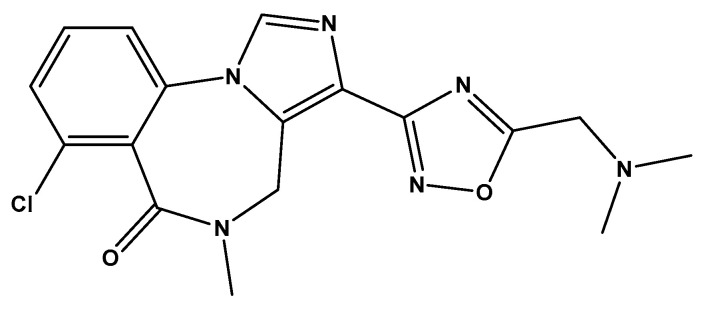
Structure of dimdazenil.

In November 2023, dimdazenil capsules was approved by the NMPA (National Medical Products Administration) for marketing, which had achieved breakthrough results in reducing impairment of daytime function. Dimdazenil (Figure 1) is a partial positive allosteric modulator of the γ-aminobutyric acid type A receptor that promotes sleep through partial activation of this receptor [1]. Given the significant clinical application of this drug, it is necessary to develop appropriate quality control methods to ensure its safety, efficacy and quality controllability, thereby further promoting the wide application of this drug in clinical practice. However, this preparation has a relatively high proportion of excipients and low API content (~1.5%, *w*/*w*), making the accurate determination of low-concentration API in such complex matrices a considerable challenge.

In recent years, Transmission Raman spectroscopy (TRS) has been widely applied in the quantitative analysis of active pharmaceutical ingredients (APIs) and excipients in pharmaceutical formulations of drugs and polymorphs [2,3,4,5,6,7,8,9,10,11]. This technique obviates the need for sample preparation, enables rapid and non-destructive measurements, and yields highly specific chemical information alongside accurate quantitative data from dense or highly turbid samples [3,4,5,6,7,12,13,14]. The previously mentioned characteristics of TRS make it occupy a significant position in the pharmaceutical industry and demonstrates substantial potential for future development [15,16,17,18,19]. It precisely aligns with the requirements of continuous manufacturing, particularly in terms of analytical throughput and other key aspects. Content uniformity testing methods using TRS have been approved by regulators using International Council for Harmonisation of Technical Requirements for Pharmaceuticals for Human Use (ICH) and regulatory authority-acceptable protocols [17,18,19]. Currently, while there have been reports on the use of TRS for determining drug formulation content, most studies focus on the quantitative analysis of high-concentration APIs [4,5,7,8,10,11]. In contrast, the application of TRS for determining low-concentration APIs in complex matrices remains relatively underreported [20,21]. To the best of our knowledge, no study has yet demonstrated the feasibility of TRS for accurate quantification of APIs at concentrations as low as ~1.5% *w*/*w* in commercial capsule formulations, representing a critical gap for its adoption in low-dose drug products.

In the present study, we aimed to develop a method for determining the content of dimdazenil capsules by combining TRS with the partial least squares (PLS) method for cost-efficient and fast at-line analysis in continuous manufacturing processes. A dimdazenil capsule content prediction model was established using calibration samples prepared based on the design of experiments (DoE) approach and calibrated using high-performance liquid chromatography (HPLC) results. API contents in batch-produced dimdazenil capsules were predicted using the model and compared with the corresponding HPLC measurements. The development of faster and more effective analytical methods will undoubtedly contribute to more efficient, streamlined, and cost-effective pharmaceutical manufacturing processes compared to HPLC.

## 2. Materials and Methods

In alignment with the identified gaps in low-concentration API quantification, we employed a DoE-based approach to introduce variability and develop a robust PLS model.

### 2.1. Materials

The following reagents and test drugs were used in this study: ammonium acetate (analytical reagent grade, batch number: 20160728, purity ≥ 98.0%, Sinopharm Chemical Reagent Co., Ltd., Shanghai, China), acetic acid (guaranteed reagent grade, batch number: 20220121, purity ≥ 99.8%, Sinopharm Chemical Reagent Co., Ltd., Shanghai, China), acetonitrile (liquid chromatography grade, batch number: X8GA1H, purity: 99.999%, Honeywell, Muskegon, MI, USA), and three batches of dimdazenil capsules (batch numbers: A22051301, A22051401, and A22050701, Zhejiang Jingxin Pharmaceutical Co., Ltd., Shaoxing, China).

### 2.2. Instruments

The following instruments were used in this study: a transmission Raman spectrometer (TRS100, Agilent, London, UK) with ContentQC spectra acquisition software (version 3.4.6.8, Agilent, London, UK) and Solo data processing software (version 8.6.2, Eigenvector Research Inc., Manson, WA, USA), an HPLC system (LC-2040C, Shimadzu, Kyoto, Japan) with LabSolutions software (version 6.90 SP2, Shimadzu, Kyoto, Japan), electronic balances (XS205DU, Mettler Toledo, Greifensee, Switzerland; YP3002N, Shanghai Jinghai Instruments, Shanghai, China), a pH meter (S475, Mettler Toledo, Greifensee, Switzerland), water purification system (Milli-Q, Millipore, Billerica, MA, USA), and an ultrasonic cleaner with LED display (KS-600DE, Kunshan Jielimei Ultrasonic Instrument, Kunshan, China).

### 2.3. HPLC Conditions

HPLC was performed using a Kromasil 100-5-C18 column (4.6 × 150 mm, 5 μm) and acetate buffer (pH 5.8)–water–acetonitrile (10:65:25) as the mobile phase. The acetate buffer solution was obtained by dissolving 3.85 g ammonium acetate in 500 mL of water and adjusting the pH to 5.8 using acetic acid. The operating parameters were as follows: detection wavelength: 250 nm; column temperature: 30 °C; injection volume: 20 μL. Sample solutions were prepared as follows: one capsule was placed in a 100 mL volumetric flask, and an appropriate volume of the mobile phase was added to the flask. After sonication of the flask contents for 30 min (power: 50 W; frequency: 40 kHz) until the occurrence of capsule disintegration, the solution was allowed to cool down to room temperature, made up to the mark, and shaken uniformly to obtain the sample solution.

### 2.4. TRS Acquisition Parameters

The API and excipients were portioned into 7.5 cm^2^ transparent plastic bags and scanned by TRS to obtain optimized scanning parameters (lens collection aperture: medium; laser spot diameter: 4.0 mm; laser power: 0.65 W; exposure time: 5 s; number of accumulations: 20). Subsequently, the dimdazenil capsules were scanned and the characteristic peaks of dimdazenil were identified.

### 2.5. Sample Preparation

The dimdazenil capsules used in this study were composed of dimdazenil (1.51% *w*/*w*), excipient A (50.75% *w*/*w*), excipient B (29.00% *w*/*w*), and other excipients (C/D/E) (18.74% *w*/*w*). Simple formulations with low API concentrations including 15 formulation levels (5 levels × 3 main components) were established using the central composite design (CCD) DoE method. The components were varied independently, resulting in a stable design space centered around the target product.

For sample preparation, we used excipients and capsule shells from the same suppliers as those of the production batches. Dimdazenil and excipients of the same batch were mixed uniformly and filled into capsule shells of the same batch to prepare capsules of identical form, size, and shape as those in the production batches. Table 1 shows the formulation levels determined by DoE, with fill weight controlled around 165.53 ± 5 mg (160.53–170.53 mg). Calibration and validation samples were prepared simultaneously. In total, 25 capsules (20 calibration samples and 5 validation samples) were prepared for each formulation level, and the actual fill weights were recorded.

### 2.6. Sample Scanning

The calibration samples were scanned to obtain 300 raw spectra, where each individual spectrum (from 15 formulation levels × 20 capsules per level) is assigned a unique color. Using Solo data processing software, the spectra were loaded into the model as the X module, and % *w*/*w* data were loaded into the model as the Y module. The raw spectra were baseline-corrected and normalized to reveal spectral trends in function of the dimdazenil concentration.

### 2.7. PLS Model Establishment

Chemometric analysis and model development were performed using Solo software For the construction of the PLS quantitative model to assay dimdazenil, fifteen concentration levels of capsules were selected, with 20 capsules at each concentration level, and each capsule was scanned once. The Raman spectra of these samples served as the input variables (X), while the drug content determined by HPLC was used as the response variable (Y). All spectra were preprocessed with baseline correction and standard normal variate (SNV), followed by mean-centering of the variables, before being imported into Solo software to build the calibration model.

The predicted drug content values (ŷ_i) were compared against the actual HPLC-measured values (y_i), where n denotes the number of samples in the prediction set. The Root Mean Square Error of Calibration (RMSEC) and Cross-Validation (RMSECV) were calculated as described in references [10,11]. Specifically, RMSEC was computed using the predicted values from the calibration set. RMSECV was derived from the predicted values obtained via cross-validation (using the Venetian blinds method) applied to the calibration set.

To evaluate the performance of the developed model, it was used to predict the API content in real capsules. The model, built using HPLC results as reference values and TRS predictions as response values, was validated accordingly. A total of 15 concentration levels were included in the validation, with five capsules scanned per level using TRS, resulting in 75 validation samples. The predicted contents from TRS were compared against the corresponding HPLC-measured values to assess the accuracy and feasibility of the model.

### 2.8. Commercial Batch Scanning

We selected three individual capsules from batch A22050701 and performed six replicate TRS measurements on each. The feasibility of the established model was further validated using three batches of dimdazenil capsules. Five capsules from each batch were scanned by TRS to obtain 15 spectra. To assess the robustness of the method in a routine QC environment, these quantitative analyses were performed by different analysts on different days.

## 3. Results

### 3.1. Method Feasibility

**Figure 2:**
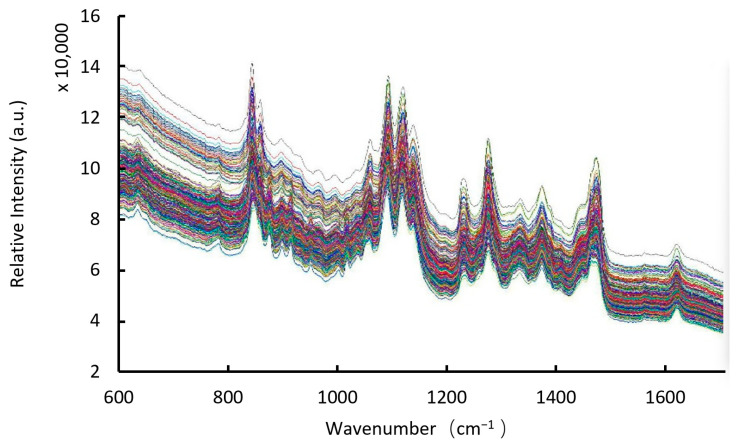
300 raw spectra of calibration samples (600 cm^−1^ to 1710 cm^−1^. The spectra of different colors were obtained through different scans).

**Figure 3:**
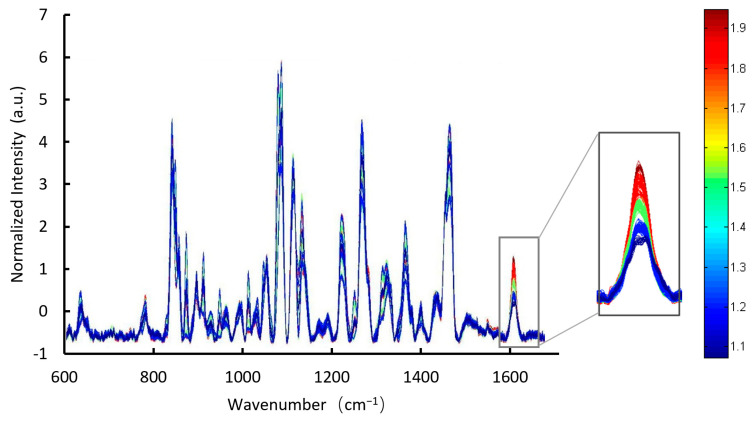
300 baselined and normalized spectra of calibration samples (600 cm^−1^ to 1710 cm^−1^). The inset shows a magnification of the 300 spectra at 1628 cm, with different colors denoting different dimdazenil concentrations (ranging from blue for low concentrations to red for high concentrations).

**Figure 4:**
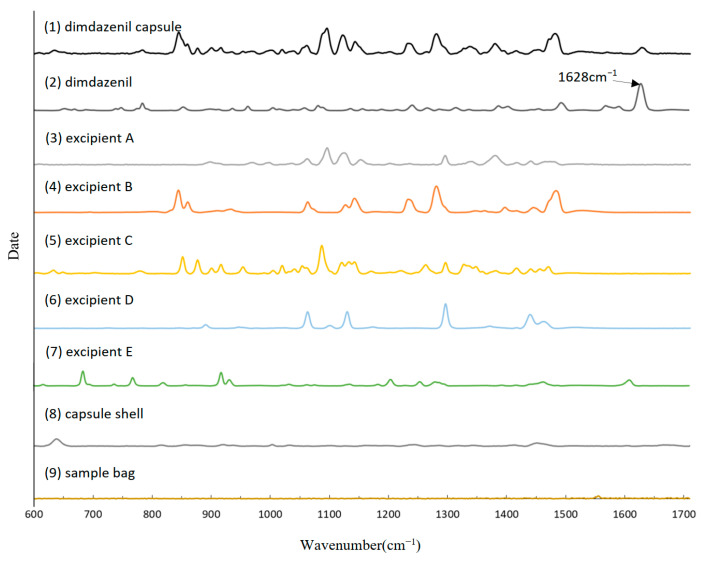
Baselined and normalized spectra of various components.

First, we collected the TRS spectrum of the dimdazenil capsule (Figure 2, Figure 3 and Figure 4). As shown in Figure 4, the transmission Raman spectra of the individual formulation components reveal that the signal from intact capsules is overwhelmingly dominated by Excipient B and Excipient C. This is consistent with their high mass fractions in the blend. Owing to the large powder fill weight per capsule and the inherent volumetric sensitivity of TRS, spectral contributions from the capsule shell and plastic sample bag are negligible under the current measurement conditions. As shown in Table 2:

- The medium-intensity band at 1609 cm^−1^ arises from C=C stretching vibrations of the aromatic ring in Excipient E;
- The intense doublet at 1468 cm^−1^ and 1441 cm^−1^ arises exclusively from CH_2_ scissoring vibrations of the abundant methylene groups in Excipient B;
- The very strong and broad band centered at 1125 cm^−1^ is attributed to asymmetric C–O–C ether stretching vibrations within the triblock copolymer structure of Excipient B;
- The sharp band at 1088 cm^−1^ results from coupled C–O–C and C–C skeletal vibrations of Excipient B;
- The characteristic bands at 1052 cm^−1^ and 940 cm^−1^ are due to C–O stretching and crystalline modes of Excipient C, respectively.

Although Excipient E shows a medium-intensity Raman band at 1609 cm^−1^ in pure form, its extremely low level in the final capsule (0.01% of API content) renders this signal negligible, and no contribution is observed at 1628 cm^−1^ post-preprocessing, confirming no interference with quantification. In sharp contrast, the intense and well-resolved peak at 1628 cm^−1^ constitutes the only diagnostic band of dimdazenil detectable in the intact capsule. This peak originates from strongly coupled C=N and C=C stretching vibrations of the extended conjugated system. No excipient produces any Raman scattering at this wavenumber, thereby ensuring absolute specificity. Accordingly, the 1628 cm^−1^ band represents the sole reliable, selective, and quantitative marker for non-destructive identification and content/content-uniformity determination of dimdazenil capsules using TRS [22].

**Table 2:** Dimdazenil capsule Raman spectroscopy characteristic peak component attribution table.

Wavenumber (cm^−1^)	Dimdazenil (%)
1628	Dimdazenil
1609	Excipient E
1468	Excipient B
1441	Excipient B
1125	Excipient B
1088	Excipient B
1052	Excipient C
940	Excipient C
840	Excipient B

### 3.2. Model Validation

**Figure 5:**
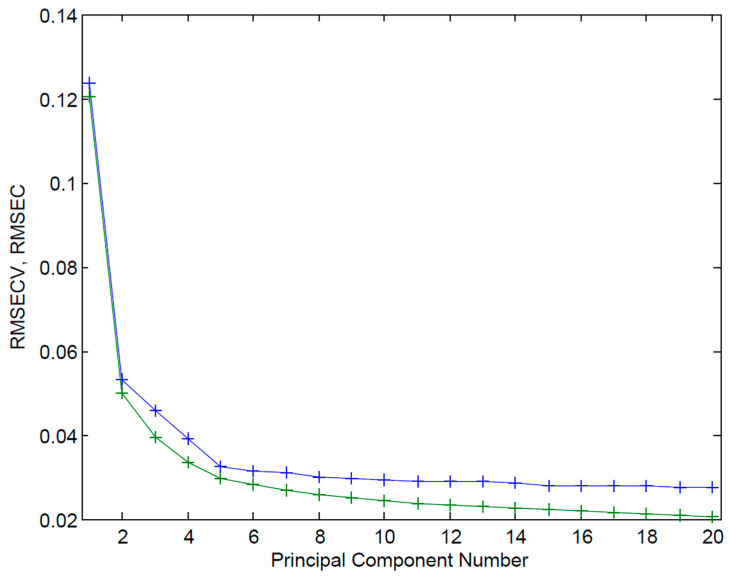
RMSEC (green plus signs, solid line) and RMSECV (blue plus signs, solid line) as a function of the number of PCA factors (1–20).

The optimal number of principal component analysis (PCA) factors was determined using three criteria: RMSECV, the unexplained variance (Q-residual), and the preservation of chemically meaningful spectral features. Q-residuals decreased progressively with increasing factors, 28.85% (1 factor), 4.91% (2), 2.98% (3), 2.14% (4), 1.73% (5), and 1.48% (6), indicating that five factors already captured over 98% of the total spectral variance. With five factors, RMSEC and RMSECV were 0.02963 and 0.03253 (Figure 5), which represented the smallest difference between the two errors across all models tested and showed no evidence of overfitting. Examination of regression coefficient profiles for models with six or more factors showed a clear loss of the characteristic absorption band at 1628 cm^−1^, replaced by high-frequency noise across the spectrum. This indicates that additional factors beyond five began to fit non-chemical variations rather than meaningful analyte signals. To ensure that the model-predicted results are close to the actual values, theoretical API concentrations based on the model (Table 1) were replaced with the actual mass of each capsule component as measured by HPLC, and the model was corrected after parameter optimization. Subsequent to this correction, the five-factor model remained the optimal selection.

Upon transitioning to PLS mode, the software automatically inherited the preprocessing protocols established during PCA for model recalculation. While an initial number of Latent Variables (LVs) was suggested by internal cross-validation, models were systematically constructed with LV counts ranging from 2 to 6 to ensure the selection of an optimal configuration. By comparing the Root Mean Square Error of Prediction (RMSEP) and prediction bias across these distinct models, the 5-LV model was identified as the optimal solution.

The validation results, as summarized in Table S1, demonstrate the strong predictive performance of the TRS model. The RMSEP was 0.0513 mg, accompanied by a low prediction bias of 0.0148 mg, indicating good agreement between TRS predictions and HPLC reference values across the validation set (*n* = 75). The relative errors were predominantly maintained within a 5% threshold, yielding a mean relative error of 1.60% (ranging from 0.02% to 4.80%). Instead of significant systematic deviation, the method showed high accuracy with a mean recovery of 100.5% and a negligible mean difference of 0.0145 mg between the TRS and HPLC measurements. Consequently, these findings suggest that the TRS model possesses sufficient predictive accuracy to serve as a practical alternative for rapid API content quantification within the specified range.

**Figure 6:**
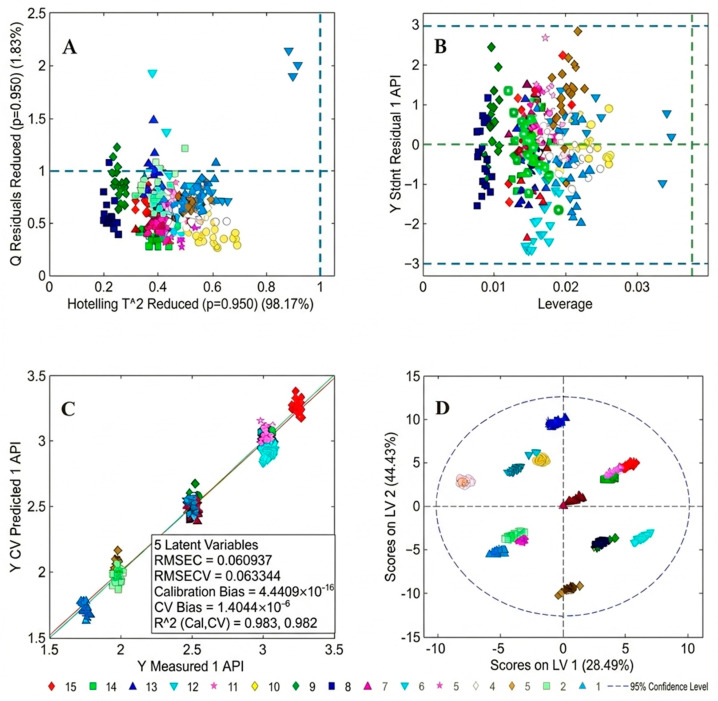
(**A**) Hotelling T^2^ vs. Q-residuals plot, where dashed lines indicate the 95% confidence limits (*p* = 0.95) for outlier detection; (**B**) Studentized residuals vs. leverage plot, with horizontal and vertical dashed lines representing the outlier thresholds for Y-residuals and high-influence samples, respectively; (**C**) Plot of measured vs. predicted API values for calibration and cross-validation sets; (**D**) Score plot of the first two latent variables (LVs), with the dashed ellipse representing the 95% confidence region (Hotelling’s T^2^ ellipse). The PLS model was developed using 15 formulation levels listed in Table S1.

Figure 6 shows the model established with the 15 formulation levels. Hotelling’s model and Q-residuals were employed to assess the degree of data dispersion and outliers to judge model performance for the calibration samples and sample quality. As shown in Figure 6A, the majority of samples were within the lower statistical threshold, whereas three samples (belonging to formulation level 6 in Table 1 and indicated in blue in the figure) were located far from the other samples. In the residuals vs. leverage plot in Figure 6B drawn to determine whether these were outliers, the three samples are still located far away from the other samples of the same level. The outliers observed at Formulation Level 6 (Figure 6A,B) may be associated with physical heterogeneity within individual capsules. Given the low API concentration (1.5% *w*/*w*), minor variations in API distribution—such as micro-scale inhomogeneity or localized agglomeration—within the TRS sampling volume could contribute to spectral deviations. Additionally, differences in powder packing density, potentially arising from the manual capsule filling process, might influence photon scattering behavior by altering sample density and optical properties. These factors could collectively affect model performance, leading to elevated residual values in the PLS regression. These factors represent plausible explanations for the observed outliers, though dedicated homogeneity studies would be required to elucidate their individual contributions. However, Figure 6D shows that all 300 samples were located within the 95% confidence interval, with samples of the level (indicated in the same color) being close to each other. This indicated that there was no need for outlier exclusion and demonstrated that the model was acceptable.

The RMSEC and RMSECV values and the coefficient of determination (R^2^) of the calibration model were used to judge the suitability of the established model. Figure 6C shows the linear fit between the actual measured values of the samples and the model-generated predicted values. The R^2^ value provides a measure of the ability of the model to adequately correlate the spectra with the given concentrations, with R^2^ values > 0.95 typically considered good. The R^2^ value of the model was 0.982, demonstrating its ability to correlate the spectra with the actual concentrations. The RMSEC and RMSECV values were 0.060937 mg and 0.063344 mg, respectively, which are close, indicating that the model was capable of stable sample prediction in the absence of calibration samples. The low RMSEC and RMSECV values and good linearity demonstrated the suitability of our model. Therefore, five PLS factors were selected as the optimal balance between predictive performance, model simplicity, and chemical interpretability.

### 3.3. Method Precision and Validation with Commercial Batches

#### 3.3.1. Statistical Characterization of Method Performance

The stability of the TRS model was first evaluated through repeatability studies on individual capsules from batch A22050701. As shown in Table 3, the Relative Standard Deviation (RSD) values for three separate capsules, each scanned six times, ranged from 1.37% to 1.77%, with an overall average RSD of 1.67%. This high level of reproducibility across repeated measurements confirms the method’s inherent stability.

To assess the agreement between TRS predictions and HPLC reference measurements, samples from three commercial batches (A22051301, A22051401, and A22050701) were analyzed using both methods. Table 4 presents the paired measurements, while Table 5 summarizes the batch-specific statistics.

Bland–Altman analysis was performed to quantitatively assess the agreement between TRS and HPLC methods. The analysis revealed a systematic negative bias of −2.87% (95% CI: −3.57% to −2.17%), indicating that TRS consistently underestimates API content relative to HPLC. The 95% limits of agreement (−5.57% to −0.17%) demonstrate acceptable method concordance for pharmaceutical quality control, with 14 of 15 samples (93.3%) falling within these limits. All samples (100%) met the ±5% relative error criterion. The consistent underestimation probably comes from matrix effects and excipient signals, which are common in non-destructive spectroscopy.

Despite the observed negative bias, the TRS method demonstrated precision comparable to the reference method. As summarized in Table 5, the overall RSD for TRS predictions was 1.45%, closely aligning with the 1.21% RSD observed for HPLC measurements. This indicates that the TRS model captures the variation in API content with similar reliability to the standard method.

#### 3.3.2. Advantages, Limitations, and Model Expandability

TRS offers significant operational advantages over HPLC by eliminating intensive sample pretreatment, streamlining the workflow from 30 min to under 150 s per unit, and enabling a 10-fold throughput increase. Its non-destructive nature preserves dosage form integrity, reduces waste, and aligns with ICH Q13 principles for continuous manufacturing, facilitating a shift to high-frequency in-process monitoring. Despite a systematic negative bias of −2.87%, the method shows precision (RSD 1.45%) comparable to HPLC (1.21%), with all samples meeting ±5% relative error. This bias, likely from excipient interference, can be addressed through iterative calibration. TRS excels in high-throughput at-line Process Analytical Technology (PAT) for 100% inspection and real-time quality verification within a Quality-by-Design (QbD) framework, though HPLC remains standard for absolute accuracy in batch release.

The excipients and capsule shells used for model establishment were obtained from the same suppliers as the production batches, and the batch of dimdazenil raw material was the same as that used in the production batches. For raw material, excipients, or capsule shells prepared using other processes or from other suppliers, the substances should be re-scanned to adapt the model developed in this study.

**Table 6:** ICH comprehensive validation summary table.

Parameter	ICH Q2(R2) Acceptance Criteria	Experimental Results
Specificity	No interference from excipients at analytical wavelength	Dimdazenil characteristic band at 1628 cm^−1^ No excipient bands overlap this region
Linearity	R^2^ ≥ 0.95	R^2^ = 0.98
Range	80–120% of nominal concentration	70–130% (1.06–1.96% (*w*/*w*)) of nominal concentration validated
Accuracy (Trueness)	Mean recovery of 95–105%; relative error ≤ 5%	Mean recovery of 100.5% (individual recoveries of 95.2–104.3%, n = 75, Table S1); mean bias of +0.42%; mean relative error of 1.60% (range of 0.02–4.80%); all ≤5%.
Precision Repeatability	RSD ≤ 2% for 6 replicates under same conditions.	RSD of 1.37–1.77% (n = 6 replicates per capsule, Table 3); overall mean RSD of 1.67% for batch A22050701.
Precision Intermediate	RSD ≤ 4%	RSD: 1.45% (3 operators, 3 days, 15 samples); within-batch RSD: 0.97–2.06%

### 3.4. Greenness Assessment

**Figure 7:**
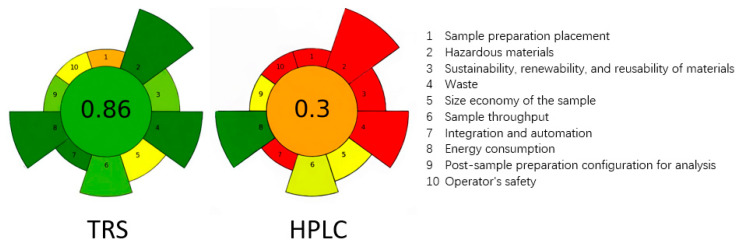
Greenness assessment by AGREEprep. The colors represent the performance level: dark green indicates excellent/high scores, yellow/orange indicates moderate performance, and red indicates poor performance/low scores.

To quantitatively evaluate the greenness of the developed TRS method, we applied the AGREEprep tool to assess the sample preparation stage. As shown in Figure 7, this solvent-free and pretreatment-free approach achieved an overall greenness score of 0.86, with predominantly green sectors across the ten criteria, reflecting minimal environmental impact. In contrast, the conventional HPLC reference method, which requires organic solvent dissolution, volume adjustment, and waste liquid generation, received a significantly lower AGREEprep score of approximately 0.3, with several red and orange sectors indicating higher environmental burden. This comparison clearly demonstrates the substantial advantages of the TRS method in eliminating solvent consumption, reducing waste generation, and lowering energy demands, thereby aligning more closely with the principles of sustainable pharmaceutical analysis.

## 4. Conclusions

In light of the precision and validation results from commercial batches, which demonstrated a mean RSD of 1.45% and relative errors below 5%, TRS offers a viable alternative to HPLC despite a minor systematic bias. Dimdazenil’s potent central nervous system effects make it essential to minimize direct exposure to operators and the environment throughout production. Although HPLC-based content uniformity testing is mandated in both the Chinese and US Pharmacopoeias, it comes with notable limitations, such as the need to handle dimdazenil solutions during sample preparation, which creates significant health risks for lab personnel.

In contrast, this study demonstrates the feasibility of using TRS as a rapid, non-destructive alternative for content variation monitoring of dimdazenil in solid dosage forms. The TRS-based method enables analysis of an entire batch in less than 10 min—significantly reducing analytical time, minimizing operator exposure to hazardous substances, and eliminating the need for solvents or chemical reagents. This high-throughput approach is particularly valuable for continuous manufacturing, allowing for rapid, large-scale quantification of dimdazenil, real-time monitoring of quality variations, and timely feedback for process parameter adjustments.

The method is especially effective for determining capsule content with low API concentrations despite interference from diverse and high-content excipients, thereby expanding TRS applicability in continuous processes. Beyond final product release testing, TRS’s rapid measurement capability positions it as a powerful enabler of PAT in continuous pharmaceutical manufacturing. While real-time in-line monitoring of dynamic processes (e.g., powder blending) remains challenging due to limitations in optical access and representative sampling, the methodology is ideally suited for high-throughput at-line applications, such as rapid, non-destructive quantification of withdrawn blended powder or capsule samples without solvent use or extensive preparation.

Compared to conventional chromatography, this approach offers superior speed, operational simplicity, and alignment with green analytical chemistry principles. The demonstrated performance supports compliance with regulatory frameworks such as the U.S. Food and Drug Administration (FDA)’s PAT initiative(2004) [23], European Medicines Agency (EMA)’s continuous manufacturing guidelines(2022) [24], and ICH Q13, thereby facilitating a QbD-driven control strategy. Getting to full in-line integration will still take some engineering work to sort out interface stability and flow dynamics. However, the analytical framework we have built here lays the essential groundwork to make that transition possible.

Although we saw a small systematic bias of –2.87% in the commercial batches, the method still performs well enough on specificity, accuracy, precision, linearity, and range to meet ICH Q2(R2) expectations for at-line process monitoring, as summarized in Table 6. With more calibration data, it could be even tighter. The consistent underestimation probably comes from matrix effects and excipient signals, which are common in non-destructive spectroscopy. Furthermore, this method provides a valuable reference for the continuous manufacturing of dimdazenil capsules.

## Figures and Tables

**Table 1:** Capsule formulations. (% *w*/*w* for each of the samples).

Formulation Level	Dimdazenil (%)	Excipient A (%)	Excipient B (%)	Other Excipients (%)	Total Weight (%)
01	1.06	50.75	29.00	19.20	100.0
02	1.19	61.40	22.91	14.50	100.0
03	1.19	61.40	35.09	2.32	100.0
04	1.19	40.09	22.91	35.81	100.0
05	1.19	40.09	35.09	23.63	100.0
06	1.51	50.75	20.30	27.45	100.0
07	1.51	50.75	29.00	18.75	100.0
08	1.51	50.75	37.70	10.05	100.0
09	1.51	65.97	29.00	3.52	100.0
10	1.51	35.52	29.00	33.97	100.0
11	1.83	61.40	22.91	13.86	100.0
12	1.83	61.40	35.09	1.68	100.0
13	1.83	40.09	22.91	35.17	100.0
14	1.83	40.09	35.09	23.00	100.0
15	1.96	50.75	29.00	18.29	100.0

**Table 3:** Repeatability of TRS measurements on individual capsules (batch A22050701).

Capsule No.	Measurement						Mean (%)	RSD (%)
	1	2	3	4	5	6		
1	98.83	99.67	97.85	101.13	98.00	102.23	99.62	1.77
2	98.39	99.89	96.14	99.05	97.72	96.04	97.87	1.59
3	98.49	97.75	99.29	100.78	97.80	97.02	98.52	1.37
Overall							98.67	1.67

**Table 4:** Comparison of HPLC-measured API and TRS-predicted contents in samples from three production batches.

Batch No.	Sample No.	HPLC (%)	TRS (%)	Relative Error (%)
A22051301	1	98.61	96.95	1.68
	2	100.50	98.56	1.93
	3	98.86	96.36	2.53
	4	99.93	97.50	2.43
	5	97.20	96.25	0.98
A22051401	1	97.47	97.80	0.34
	2	99.74	96.09	3.66
	3	98.89	95.53	3.40
	4	99.80	96.46	3.35
	5	99.53	95.76	3.79
A22050701	1	99.72	96.32	3.42
	2	99.60	94.69	4.93
	3	101.59	97.97	3.56
	4	99.98	96.71	3.27
	5	97.46	92.88	4.70

**Table 5:** Comparison of the mean values and RSDs between HPLC-measured API content and TRS-predicted content.

Batch No.	HPLC		TRS	
	Mean (%)	RSD (%)	Mean (%)	RSD (%)
A22051301	99.02	1.29	97.12	0.97
A22051401	99.09	0.98	96.33	0.93
A22050701	99.67	1.48	95.71	2.06
Overall	99.26	1.21	96.39	1.45

## Data Availability

The original contributions presented in this study are included in the article/Supplementary Materials. Further inquiries can be directed to the corresponding author(s).

## Supplementary Materials

The following supporting information can be downloaded at https://www.mdpi.com/article/10.3390/pharmaceutics18030298/s1. Table S1: Comparison of TRS-predicted and HPLC-measured API contents in validation samples.
