# Supplementary material for: Cost-Efficient and Fast At-Line Assessment of Content and Uniformity in Low-Dose Dimdazenil Capsules Using Transmission Raman Spectroscopy
Source: Pharmaceutics. 2026 Feb 27;18(3):298. doi: 10.3390/pharmaceutics18030298 (PMC13028709; doi:10.3390/pharmaceutics18030298)
Supplement: Supplementary file 1 [file pharmaceutics-18-00298-s001.zip › pharmaceutics-4087028-supplementary.pdf]

Supplementary Material

Cost-Efficient and Fast At-line Assessment of Content and Uniformity in Low-Dose Dimdazenil Capsules Using Transmission Raman Spectroscopy

Xun Ma [1,†], Lianlian Shan [2,†], Shuangpeng Zhu [3], Zihan Zhu [1,4], Shuyu Lu [1,4], Mingzhe Xu [1,*] and Lihui Yin [1,*]

[1] NMPA Key Laboratory for Quality Research and Evaluation of Chemical Drugs, National Institutes for Food and Drug Control, Beijing 102629, China; maxun@nifdc.org.cn (X.M.);

[2] Xinjiang Uyghur Autonomous Region Institute for Drug Control, Urumqi 830054, China; 13579808851@163.com (L.S.);

[3] Zhejiang Jingxin Pharmaceutical Co., Ltd, Shaoxing 312500, China; zhushuangpeng@jingxinpharm.com (S.Z.);

[4] China Pharmaceutical University, Nanjing 210009, China; 3322010555@stu.cpu.edu.cn (Z.Z.); 3323010554@stu.cpu.edu.cn (S.L.);

* Correspondence: xumzhe@nifdc.org.cn (M.X.); yinlihui@nifdc.org.cn (L.Y.);

† These authors contributed equally to this work.

Table S1. Comparison of TRS-predicted and HPLC-measured API contents in validation samples（n=75）.

Formulation level	Theoretical Content(mg)	TRS (mg)	HPLC (mg)	TRS (%)	HPLC (%)	Relative error(%)	Recovery (%)
01	1.75	1.743	1.706	99.60	97.49	2.11	102.2
01	1.75	1.706	1.677	97.48	95.81	1.67	101.7
01	1.75	1.669	1.676	95.39	95.79	-0.4	99.6
01	1.75	1.701	1.663	97.19	95.03	2.16	102.3
01	1.75	1.690	1.641	96.57	93.79	2.78	103.0
02	1.975	1.800	1.845	91.14	93.40	-2.26	97.6
02	1.975	1.833	1.828	92.81	92.55	0.26	100.3
02	1.975	1.798	1.763	91.05	89.25	1.8	102.0
02	1.975	1.819	1.851	92.10	93.71	-1.61	98.3
02	1.975	1.835	1.814	92.93	91.86	1.07	101.2
03	1.975	1.866	1.908	94.47	96.60	-2.13	97.8
03	1.975	1.904	1.901	96.41	96.27	0.14	100.1
03	1.975	1.914	1.950	96.92	98.74	-1.82	98.2
03	1.975	1.882	1.879	95.28	95.13	0.15	100.2
03	1.975	1.889	1.915	95.63	96.98	-1.35	98.6
04	1.975	1.877	1.894	95.03	95.88	-0.85	99.1
04	1.975	1.850	1.820	93.66	92.14	1.52	101.6
04	1.975	1.750	1.752	88.61	88.70	-0.09	99.9

04	1.975	1.832	1.794	92.75	90.82	1.93	102.1
04	1.975	1.874	1.836	94.88	92.95	1.93	102.1
05	1.975	1.830	1.878	92.65	95.09	-2.44	97.4
05	1.975	1.825	1.905	92.40	96.46	-4.06	95.8
05	1.975	1.812	1.904	91.76	96.39	-4.63	95.2
05	1.975	1.837	1.896	93.02	96.01	-2.99	96.9
05	1.975	1.924	1.938	97.43	98.15	-0.72	99.3
06	2.5	2.240	2.268	89.61	90.72	-1.11	98.8
06	2.5	2.205	2.224	88.21	88.94	-0.73	99.2
06	2.5	2.287	2.290	91.49	91.58	-0.09	99.9
06	2.5	2.272	2.270	90.87	90.81	0.06	100.1
06	2.5	2.251	2.239	90.05	89.55	0.5	100.6
07	2.5	2.329	2.329	93.17	93.15	0.02	100.0
07	2.5	2.363	2.356	94.50	94.25	0.25	100.3
07	2.5	2.314	2.325	92.57	93.00	-0.43	99.5
07	2.5	2.376	2.366	95.05	94.62	0.43	100.5
07	2.5	2.361	2.365	94.44	94.60	-0.16	99.8
08	2.5	2.347	2.359	93.86	94.37	-0.51	99.5
08	2.5	2.360	2.340	94.40	93.58	0.82	100.9
08	2.5	2.318	2.337	92.70	93.49	-0.79	99.2
08	2.5	2.404	2.442	96.15	97.69	-1.54	98.4
08	2.5	2.369	2.368	94.76	94.73	0.03	100.0

09	2.5	2.306	2.326	92.25	93.05	-0.8	99.1
09	2.5	2.407	2.416	96.29	96.63	-0.34	99.6
09	2.5	2.457	2.478	98.28	99.13	-0.85	99.1
09	2.5	2.396	2.404	95.85	96.17	-0.32	99.7
09	2.5	2.408	2.441	96.31	97.63	-1.32	98.6
10	2.5	2.284	2.322	91.37	92.87	-1.5	98.4
10	2.5	2.298	2.332	91.90	93.26	-1.36	98.5
10	2.5	2.274	2.296	90.97	91.85	-0.88	99.0
10	2.5	2.278	2.276	91.10	91.05	0.05	100.1
10	2.5	2.311	2.353	92.44	94.10	-1.66	98.2
11	3.025	2.757	2.744	91.14	90.71	0.43	100.5
11	3.025	2.794	2.807	92.36	92.79	-0.43	99.5
11	3.025	2.827	2.800	93.44	92.55	0.89	101.0
11	3.025	2.757	2.772	91.14	91.64	-0.5	99.5
11	3.025	2.867	2.852	94.78	94.29	0.49	100.5
12	3.025	2.879	2.784	95.17	92.02	3.15	103.4
12	3.025	2.860	2.797	94.55	92.46	2.09	102.3
12	3.025	2.928	2.891	96.78	95.57	1.21	101.3
12	3.025	2.828	2.803	93.49	92.67	0.82	100.9
12	3.025	2.851	2.735	94.25	90.40	3.85	104.3
13	3.025	2.934	2.824	96.99	93.34	3.65	103.9
13	3.025	2.896	2.785	95.73	92.08	3.65	104.0

13	3.025	2.918	2.886	96.47	95.41	1.06	101.1
13	3.025	2.942	2.867	97.25	94.78	2.47	102.6
13	3.025	2.938	2.820	97.13	93.22	3.91	104.2
14	3.025	2.973	2.879	98.28	95.17	3.11	103.3
14	3.025	2.953	2.873	97.63	94.96	2.67	102.8
14	3.025	2.965	2.873	98.02	94.96	3.06	103.2
14	3.025	2.991	2.899	98.87	95.85	3.02	103.2
14	3.025	2.972	2.914	98.24	96.32	1.92	102.0
15	3.25	3.065	3.068	94.30	94.39	-0.09	99.9
15	3.25	3.133	3.017	96.41	92.83	3.58	103.9
15	3.25	3.037	2.993	93.46	92.08	1.38	101.5
15	3.25	3.078	2.986	94.70	91.87	2.83	103.1
15	3.25	3.151	3.039	96.94	93.51	3.43	103.7
Mean						1.60	100.5

RMSEP (mg)=0.0513

Bias (mg)=0.0148
